# Mitochondrial pyruvate carrier function determines cell stemness and metabolic reprogramming in cancer cells

**DOI:** 10.18632/oncotarget.18199

**Published:** 2017-05-25

**Authors:** Xiaoli Li, Gaoyang Han, Xiaoran Li, Quancheng Kan, Zhirui Fan, Yaqing Li, Yasai Ji, Jing Zhao, Mingzhi Zhang, Mantas Grigalavicius, Viktor Berge, Mariusz Adam Goscinski, Jahn M. Nesland, Zhenhe Suo

**Affiliations:** ^1^ Department of Oncology, The First Affiliated Hospital of Zhengzhou University, Zhengzhou, Henan Province, 450000, China; ^2^ Department of Pathology, The Norwegian Radium Hospital, Oslo University Hospital, Institute of Clinical Medicine, Faculty of Medicine, University of Oslo, Oslo, 0379, Norway; ^3^ Department of Thoracic Surgery, The First Affiliated Hospital of Xinxiang Medical University, Weihui City, Henan Province, 453000, China; ^4^ Department of Clinical Pharmacology, The First Affiliated Hospital of Zhengzhou University, Zhengzhou, Henan Province, 450000, China; ^5^ Department of Urology, The Norwegian Radium Hospital, Oslo University Hospital, Oslo, 0379, Norway; ^6^ Department of Surgery, The Norwegian Radium Hospital, Oslo University Hospital, Oslo, 0379, Norway

**Keywords:** MPC, OXPHOS, TCA, glycolysis, stem cell

## Abstract

One of the remarkable features of cancer cells is aerobic glycolysis, a phenomenon known as the “Warburg Effect”, in which cells rely preferentially on glycolysis instead of oxidative phosphorylation (OXPHOS) as the main energy source even in the presence of high oxygen tension. Cells with dysfunctional mitochondria are unable to generate sufficient ATP from mitochondrial OXPHOS, and then are forced to rely on glycolysis for ATP generation. Here we report our results in a prostate cancer cell line in which the mitochondrial pyruvate carrier 1 (MPC1) gene was knockout. It was discovered that the MPC1 gene knockout cells revealed a metabolism reprogramming to aerobic glycolysis with reduced ATP production, and the cells became more migratory and resistant to both chemotherapy and radiotherapy. In addition, the MPC1 knockout cells expressed significantly higher levels of the stemness markers Nanog, Hif1α, Notch1, CD44 and ALDH. To further verify the correlation of MPC gene function and cell stemness/metabolic reprogramming, MPC inhibitor UK5099 was applied in two ovarian cancer cell lines and similar results were obtained. Taken together, our results reveal that functional MPC may determine the fate of metabolic program and the stemness status of cancer cells *in vitro*.

## INTRODUCTION

There are two main pathways of glucose metabolism to generate energy for cell proliferation. Cells generally are prone to have oxidative phosphorylation (OXPHOS) as the primary pathway to produce energy in normal oxygen, which theoretically generates 36 molecules of ATP through the tricarboxylic acid (TCA) cycle. Glycolysis is the other way to convert glucose to lactate with 2 molecules of ATP production [1]. However, cancer cells often undergo glycolysis instead of mitochondrial OXPHOS, regardless of oxygen availability. This phenomenon is termed as Warburg effect, a hallmark in cancers [2].

Pyruvate is a hub metabolite for glucose, lipids and amino acids. Metabolic fate of pyruvate determines whether glycolysis is followed by OXPHOS, or by lactic fermentation [3]. More than 40 years ago it was speculated already that there was a transporter, which would conduct pyruvate across the mitochondrial inner membrane to the matrix [4–6]. These transporters were finally defined as mitochondrial pyruvate carrier 1 (MPC1) and mitochondrial pyruvate carrier 2 (MPC2) in mammals in 2012 [7, 8].

There are studies showing the association of mitochondrial function and tumor development [9, 10]. Indeed, mitochondrial function is closely related to the function of MPC complex, especially MPC1 in mammals [7, 8]. Recent molecularly targeted studies have shown that blocking MPC activity by specific inhibitors resulted in aerobic glycolysis and aggressive features in tumor cells [11, 12]. In addition, lower level of MPC protein expression has been correlated to poor survival in diverse types of tumors including lung, colon, kidney clear cell, prostate and esophagus squamous cell carcinomas [11–14]. It was also discovered that treatment with the MPC inhibitor UK5099 induced the expression of the stem cell markers Oct3/4 and NANOG in prostate cancer cells [11]. Furthermore, it was also reported that overexpression of MPC1 and MPC2 in colorectal cancer cell lines decreased cell growth in spheroids, decreased tumor size in subcutaneous xenografts, and the expressions of the stem cell markers ALDH, Lin28A, LGR5, and NANOG were suppressed in the MPC1 and MPC2 overexpression cells [13]. Collectively, the above studies indicate a role of MPC in cell stemness regulation.

In order to further explore the involvement of MPC in cancer biology, we performed MPC1 gene knockout in the murine prostate cancer cell line RM-1 with CRISPR/Cas9 technology. After performed successfully MPC1 knockout, the cells were extensive studied in consideration of migration, therapeutic sensitivities and the expression of cancer stem cell markers. It was verified that MPC1 gene knockout caused metabolism reprogramming towards Warburg effect and unregulated the cell stemness in the prostate cancer cells *in vitro*. In addition, blockade of MPC function with MPC inhibitor UK5099 in human ovarian cancer cell lines SKOV3 and OVCAR3 revealed similar results, strongly indicating a role of MPC in cell stemness regulation and metabolic reprogramming in cancer cells.

## RESULTS

### The MPC1 gene knockout cell line identification

**Figure 1:**
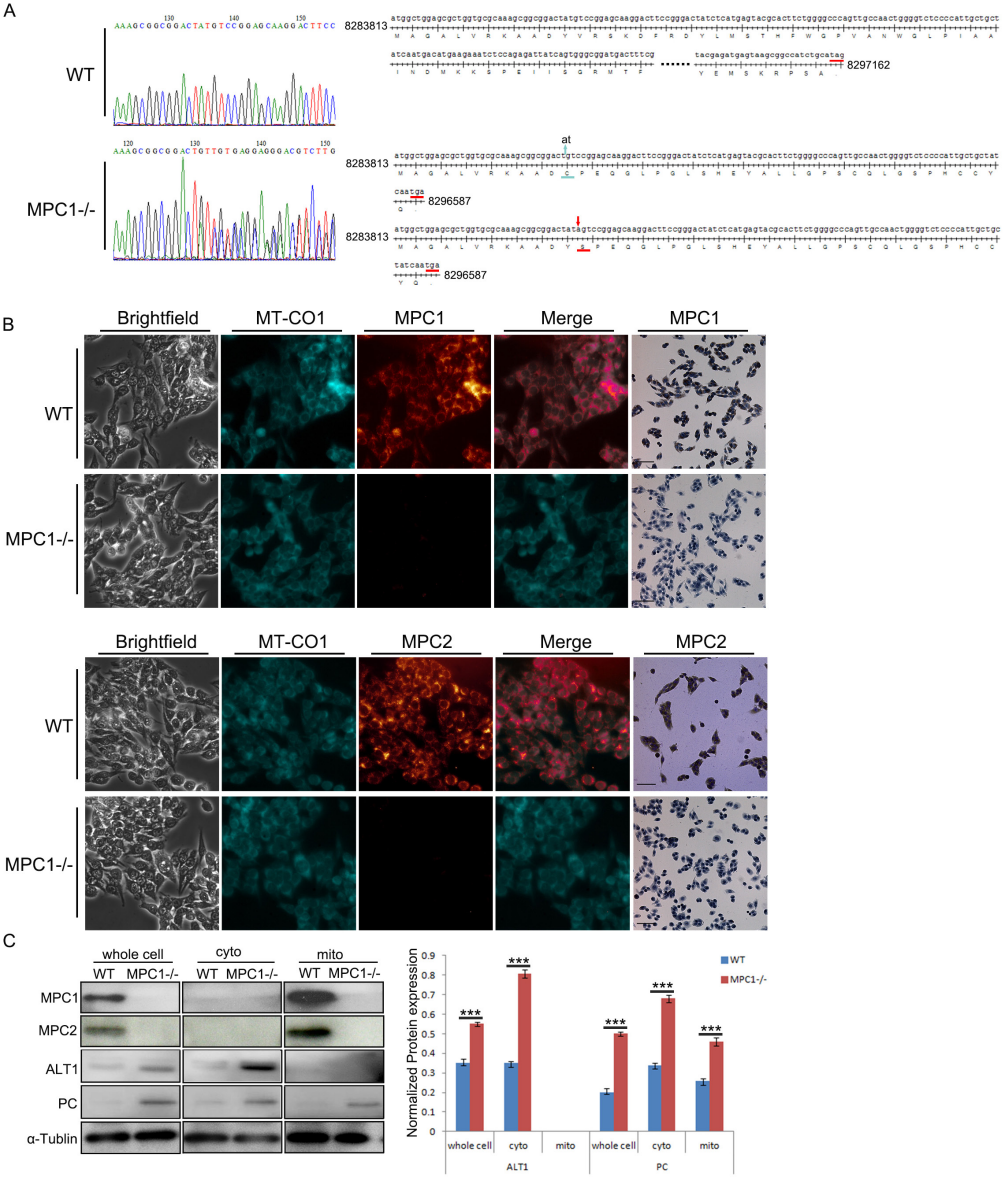
Characterization of MPC1−/− in the RM-1 murine prostate cancer cells **(A)** Shows representative sequencings of the MPC1 PCR products in WT and MPC1−/− cells. The upper panel shows a representative MPC1 PCR product sequencing chart of WT cells at the left part, and the right part on the upper panel shows the full cDNA sequence and the speculated amino acids (110 amino acids). The left part on the lower panel shows a representative MPC1 PCR product of the MPC1−/− cells, which reveals double sets of peak. The right part of the lower panel shows the mutated DNA sequences at both alleles, on which one allele on the upper part has a two-base “at” deletion (green arrow), while another allele on the lower part harbors one base “a” insertion (red arrow). Both mutated sequences create an early terminator “tga”, which results in only 1/3 of the translation (41 amino acids in the upper allele and 42 amino cods in another allele). **(B)** The IF and IHC results. Co-localization of MPC1 or MPC2 and the mitochondrial protein MT-CO1 are shown in the WT RM-1 cells, while there is no MPC1 protein expression revealed by either IF or ICC in the MPC1−/− cells. There was only weak MPC2 protein expression in the MPC1−/− cells revealed by ICC. Scale bars, 200 μm. Western blotting results of subcellular proteins from WT and MPC1−/− cells and corresponding densitometry histograms of ALT1 and PC are shown in **(C)**. Cyto means cytoplasmic protein. Mito stands for mitochondrial protein. Protein α-Tublin was used as a loading control. ***P<0.001. All experiments were performed at least three times with consistent and repeatable results.

To investigate the physiological importance of MPC1 in cancer cell proliferation and metabolism, one stable MPC1 knockout cell line (named as MPC1−/−) was established from the RM-1 cells with CRISPR/Cas9 technology. It was verified by sequencing that the MPC1−/− cell line harbored a 2bp of “at” deletion in one place as well as an “a” insertion in another place in the first exon (Figure 1A), and these alterations resulted in frameshift mutations on both alleles, which introduced a similar early terminator in the first exon in both alleles as shown in the lower right part of the Figure 1A. The immunofluorescence (IF) and immunocytochemistry (ICC) showed that MPC1 protein expression was completely disappeared in contrast to the parental cells, while the MPC2 protein expression was also reduced as shown in Figure 1B, where MT-CO1 was used as a mitochondrial locator. To further confirm the location of MPC1 and MPC2, we isolated proteins from whole cells, cytoplasm and mitochondria. Mitochondrial location of both MPC1 and MPC2 proteins was verified as shown in Figure 1C. The MPC1−/− cells expressed comparatively higher levels of pyruvate carboxylase (PC) and amino-alanine transformase1 (ALT1), which are the anaplerotic enzymes for pyruvate metabolism (Figure 1C).

### MPC1 knockout impaired oxidative TCA cycle and initiated aerobic glycolysis

**Figure 2:**
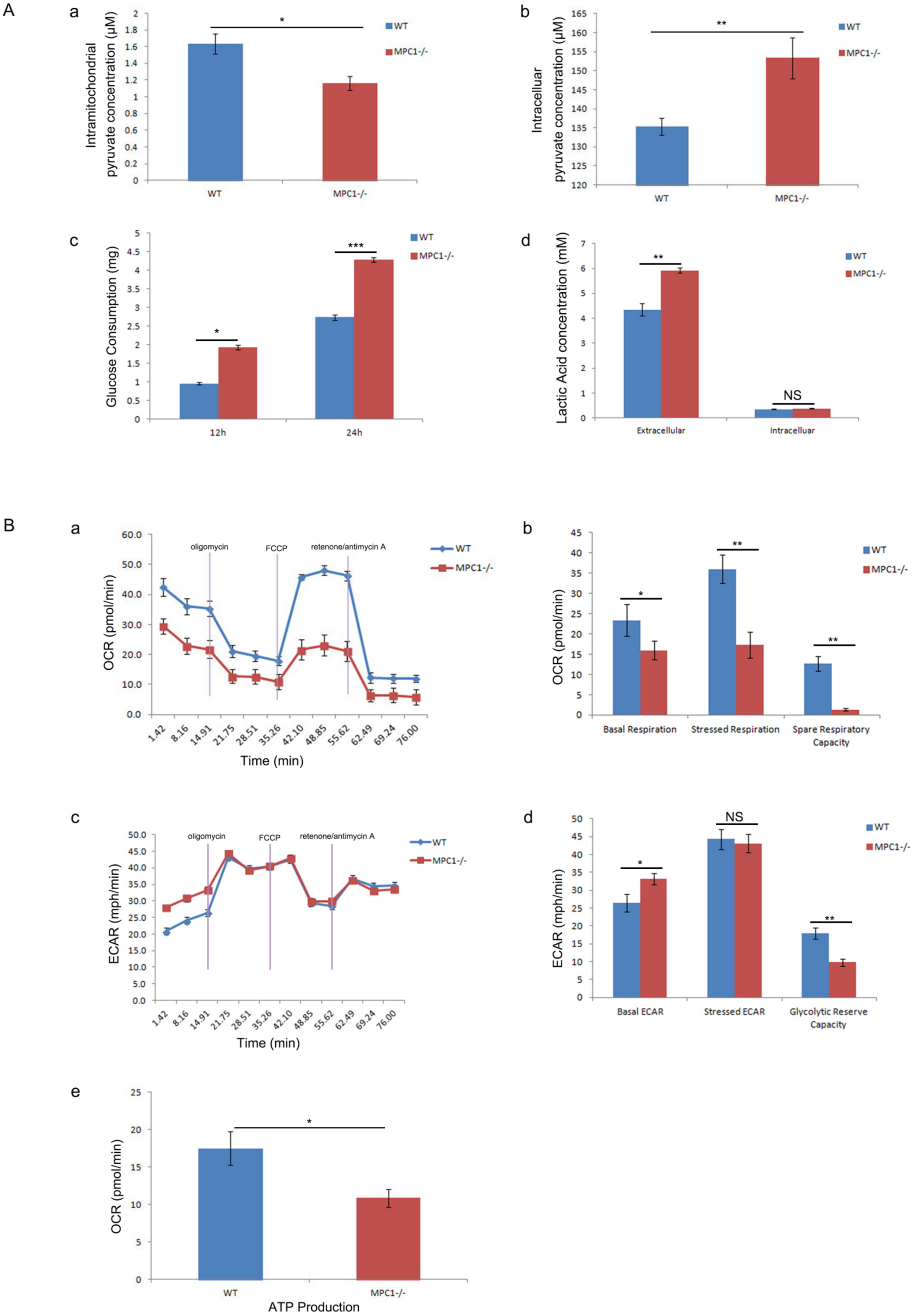
Results of mitochondrial pyruvate transportation and OXPHOS assays **(A)** Shows the results of intramitochondrial pyruvate concentration (a), intracellular pyruvate concentration (b), glucose consumption (c) and lactate concentration (d). **(B)** Shows the results of OXPHOS assays. The basic OCR and the OCR under mitostress are significantly low in the MPC1−/− cells (a). The corrsponding histograms are shown in b. The basic ECAR in the MPC1−/− cells is high (c). The spare respiratory capacity was calculated as the OCR difference between basal and stressed, and the latter was stimulated by FCCP. The histograms of ECAR (d) show significantly increased basic ECAR and significantly reduced glycolytic reserve capacity in the MPC1−/− cells, but there is no ECAR difference under stressed condition. The MPC1−/− cells generated less ATP than the WT cells as shown in e. ATP linked respiration was derived from the difference between OCR at baseline and respiration following oligomycin addition. *P<0.05, **P<0.01, ***P<0.001. Data were expressed as mean ± SEM. Three replicated experiments were carried out with the similar results.

To further confirm the functional alteration in the MPC1−/− cells, mitochondrial pyruvate concentrations in both mitochondria and cytoplasm were determined by pyruvate colorimetric assay kit. As presented in Figure 2Aa and 2Ab, the MPC1−/− cells show significantly less amount of pyruvate in the mitochondria (p=0.034), but significantly greater amount of pyruvate in the cytoplasm (p=0.006), compared to the wild type (WT) cells, indicating the blockade of pyruvate mitochondrial transportation in the cells. We next asked whether there was metabolic reprogramming in the MPC1−/− cells. Firstly, it was discovered that the MPC1−/− cells consumed significantly more glucose (p=0.03 at 12 hr, p<0.001 at 24 hr, Figure 2Ac). We then examined the difference of lactate acid secretion and cellular retention in the MPC1−/− cells. It was verified that there was significantly larger amount of extracellular lactate acid in these cells, compared to the WT cells as shown in Figure 2Ad (p=0.005). However, there was no difference in the cellular lactate acid concentration in the MPC1−/− cells in comparison to the WT cells as shown in Figure 2Ad (p=0.292).

To further reveal the status of metabolic reprogramming possibility in the MPC1−/− cells, functional mitochondria analyses were performed with seahorse XF^e^96 analyzer. It was repeatedly shown that under basal condition, cellular oxygen consumption rate (OCR) of the MPC1−/− cells was 15.98±2.86 pmol/min, which was significantly lower than that in the WT cells (23.38±3.17 pmol/min, p=0.04, Figure 2Ba and 2Bb). It was demonstrated a concomitant OCR increase (36.01±3.52 pmol/min) in the control cells, while there was only a slight OCR increase in the MPC1−/− cells (17.31±3.29 pmol/min, p=0.003, Figure 2Ba and 2Bb) under stressed condition. The injection of carbonyl cyanide-4(trifluoromethoxy) phenylhydrazone (FCCP) evoked OCR increase and demonstrated significantly reduced spare respiratory capability in the MPC1−/− cells (p=0.002, Figure 2Bb), implying that the MPC1−/− cells operated nearly maximal OCR at basal level. Low basal OCR and weak response to FCCP indicated defective mitochondrial function in the MPC1−/− cells.

Extracellular acidity rate (ECAR) is considered as an indirect analysis of the glycolytic rate of cells. The MPC1−/− cells exhibited higher basal ECAR (33.24±1.6 mpH/min) than the WT cells (26.4±2.5 mpH/min, Figure 2Bc and 2Bd, p=0.016). After injection of oligomycin, the MPC1−/− cells hold similar level of ECAR (43.1±2.5 mpH/min) as the WT cells (44.3±2.8 mpH/min, Figure 2Bc and 2Bd, p=0.609). The glycolytic reserve capacity of cells was calculated by subtracted the maximal ECAR with basal ECAR. Therefore, the MPC1−/− cells revealed significantly lower glycolytic reserve capacity, indicating that these cells operated almost maximal glycolytic rate as a compensation for the loss of OCR (Figure 2Bd, p=0.001). Cells operating mainly glycolysis usually produce less ATP. Indeed, there was significantly lower level of ATP observed in the MPC1−/− cells, compared to the WT cells as shown in Figure 2Be (p=0.011). The above results have proved that the MPC1−/− cells are forced to run aerobic glycolysis.

### MPC1 knockout resulted in activated anaplerotic pathways in the MPC1−/− cells

**Figure 3:**
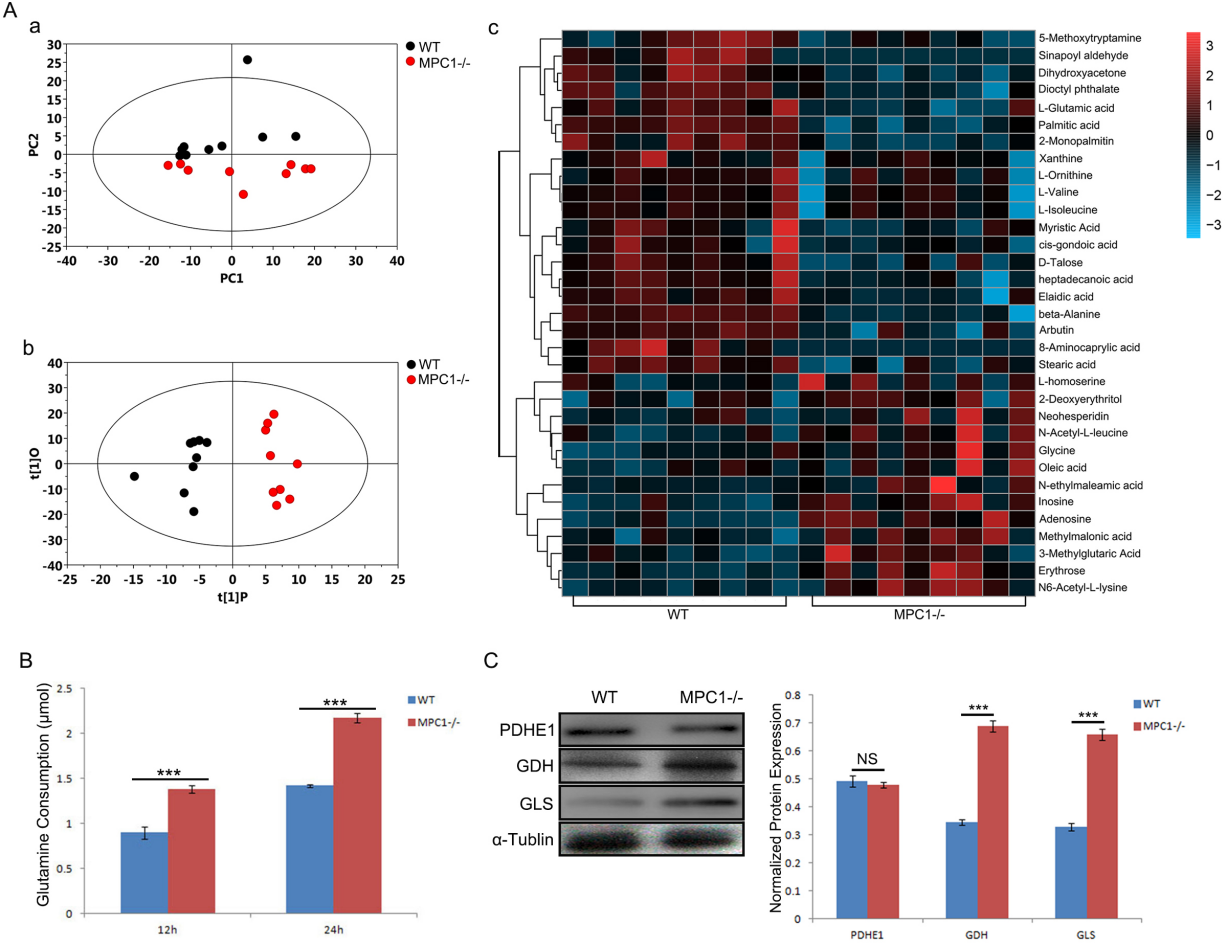
Metabolic alterations in the MPC1−/− cells **(A)** Shows PCA derived metabolite profile (a), OPLS-DA model plots (b), and the heat map analyses of the significantly altered metabolites in the MPC1−/− cells compared to the WT cells (c). Significantly more glutamine consumptions at 12 hr and 24 hr were found in the MPC1−/− cells as shown in **(B)**. All the results were normalized by cell number. MPC1−/− cells expressed significantly higher levels glutaminolysis related proteins GDH and GLS, but there was no significant change in the PDHE1 protein expression in these cells as shown in **(C)** (Representative Western blots and the corresponding histograms are shown.). The α-Tublin was used as loading control. ***P<0.001. Data were expressed as mean ± SEM. Three replicated experiments were carried out with the similar results.

To further investigate potential metabolic alterations in the MPC1−/− cells, we performed broad and steady state metabolomic analyses using Gas chromatography/mass spectrometry (GC/MS). As shown in Figure 3Aa, principal component analysis (PCA) revealed a noticeable separation between the WT and MPC1−/− cells. Supervised orthogonal projections to latent structures-discriminate analysis (OPLS-DA) were performed in this study, which can obtain a higher level of group separation and variables responsible for classification. A clear difference between the two groups was obtained as shown in Figure 3Ab. A total of 388 peak metabolites were filtrated from metabolic detection and 33 metabolites were found significantly changed as shown in Figure 3Ac (p<0.05).

It was demonstrated that the MPC1−/− cells held a 1.528-fold decrease in cellular metabolite valine and a 1.554-fold decrease in isoleucine. Moreover, alanine and L-glutamic acid were also significantly decreased in these cells (p=0.048 and p=0.049, respectively). These differences remind us that alanine and glutamine are the substrates for anaplerotic TCA cycle. As shown in Figure 3B, the MPC1−/− cells consumed significantly more glutamine both in 12 hr (p<0.001) and in 24 hr (p<0.001), indicating a strong MPC1−/− driven glutamine-anaplerosis for mitochondrial energy metabolism. To further explore the possibility of glutamine-anaplerosis, the expressions of the glutamine metabolism enzymes GLS and GDH, which convert glutamine to ketoglutarate in cells, were analyzed by Western blotting. Significantly higher levels of GLS and GDH protein expressions in the MPC1−/− cells (Figure 3C) verified the glutamine-anaplerosis in the MPC1−/− cells, although the PDHE1 protein expression is not significantly changed.

**Figure 4:**
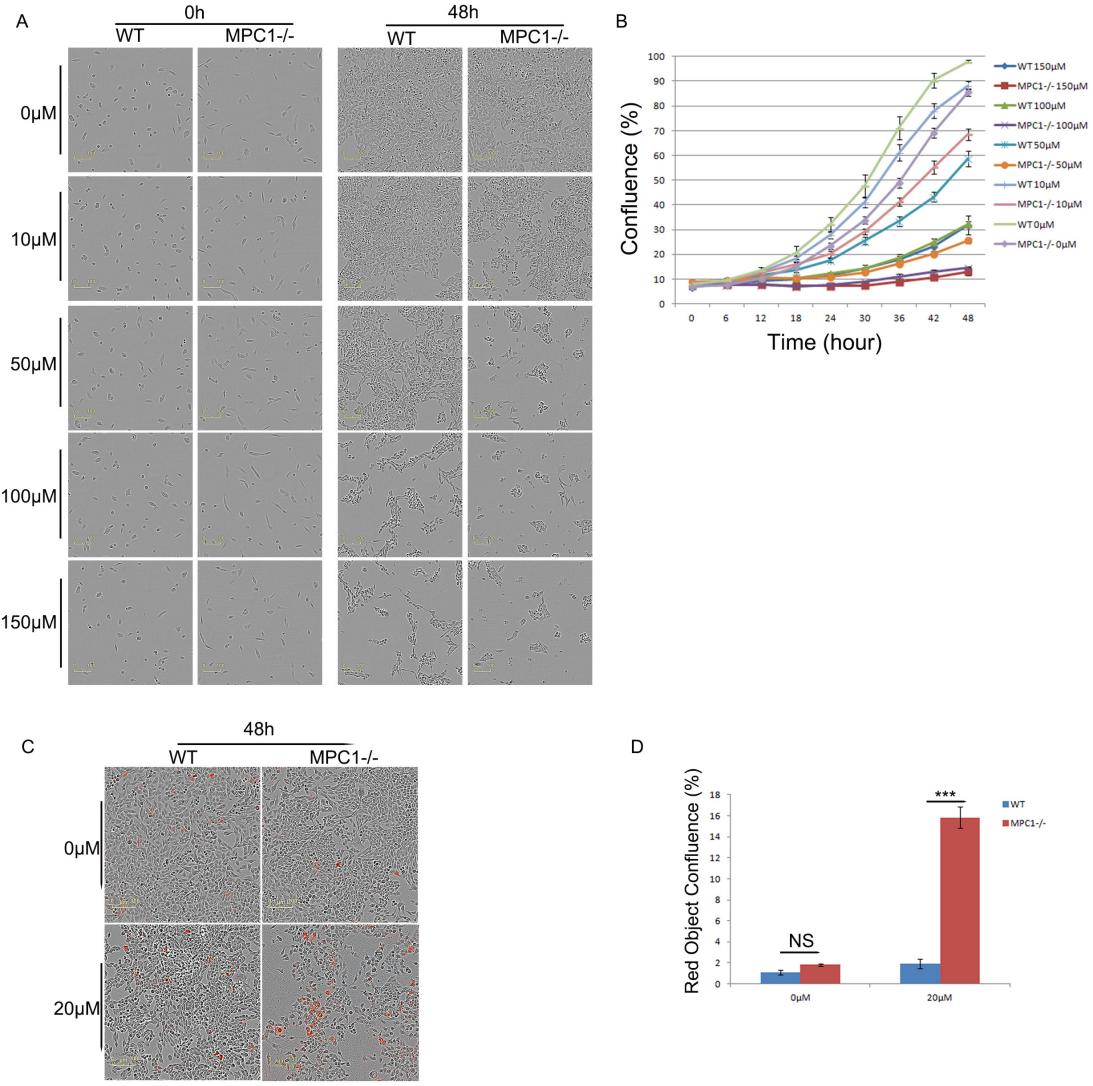
The MPC1−/− cells were more sensitive to alanine aminotransamination inhibitor β-Ch treatment Representative images obtained at 48 hr after the cells cultured in different concentrations of β-Ch and corresponding growth curves are shown in **(A)** and **(B)**, respectively. Representative images for Annexin V Red apoptosis detection and corresponding histograms of the results are shown in **(C)** and **(D)**. Scale bars, 100 μm. ***P<0.001. Data were expressed as mean ± SEM. Three replicated experiments were carried out with the similar results.

To confirm the importance of alanine, these cells were assessed for their survival ability by exposing to β-chloro-L-alanine (β-Ch), which is a competitive inhibitor of L-alanine aminotransferase (ALT) [17]. Various gradient concentrations of the inhibitor were used to treat the WT and MPC1−/− cells (10, 50, 100, 150, 250, 400, 500 μM). Along with the elevation of β-Ch concentrations, the growth ability was reduced gradually (Figure 4A and 4B). It was found that the half maximal inhibitory concentration (IC50) value for the WT cells was 64.45 μM, while IC50 value for the MPC1−/− was 20.68 μM (p=0.03). Low IC50 value for the MPC1−/− cells revealed that these cells were more sensitive to the β-Ch treatment. The MPC1−/− cells treated with 20 μM of β-Ch had significantly higher apoptosis ratio than the WT cells (p<0.001, Figure 4C and 4D).

### MPC1 knockout resulted in significantly higher levels of cellular reactive oxygen species (ROS)

**Figure 5:**
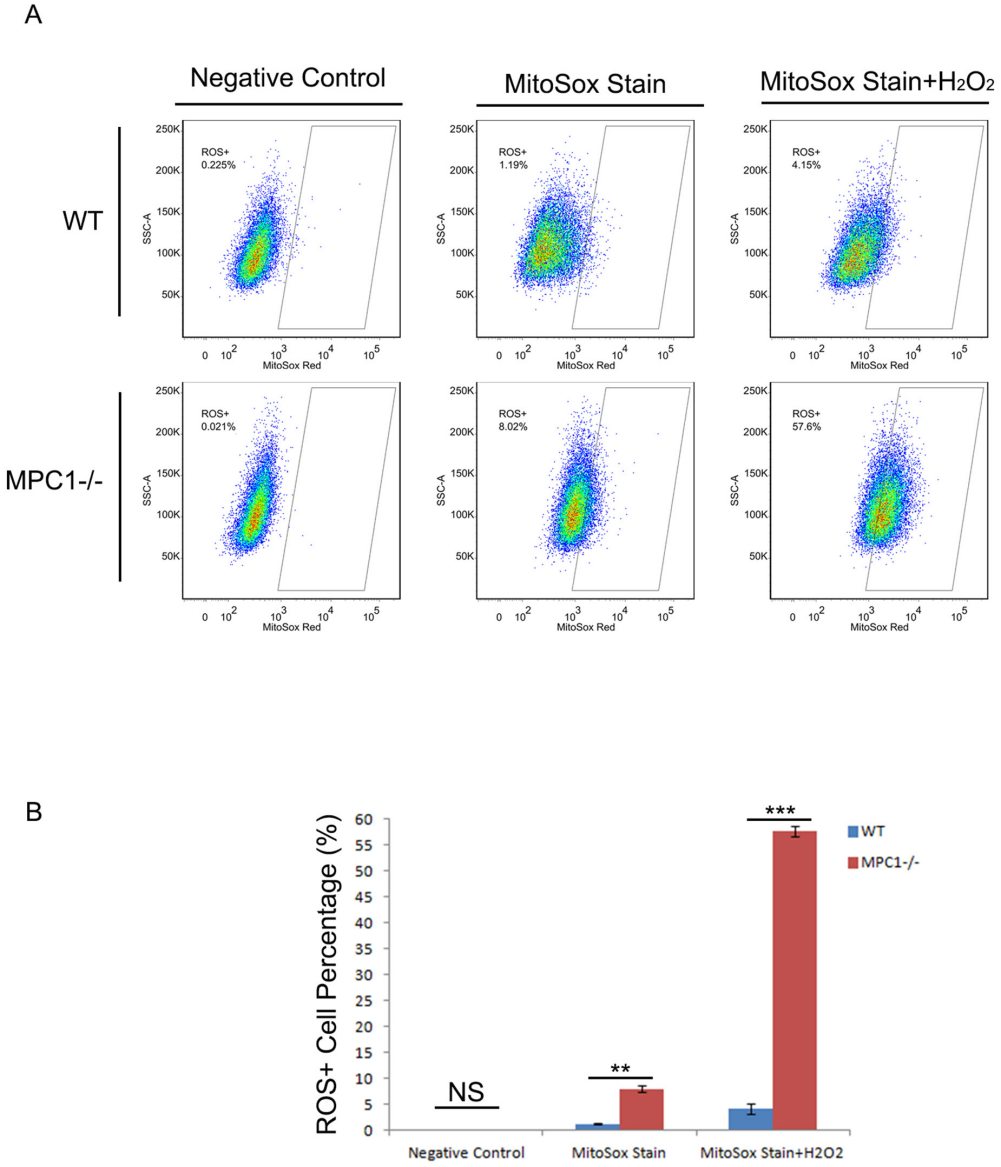
MPC1 knockout resulted in higher ROS levels Representative ROS flow cytometry images and corresponding histograms are shown in **(A)** and **(B)**, respectively. **P<0.01, ***P<0.001. Data were expressed as mean ± SEM. Three replicated experiments were carried out with the similar results.

To reveal the effect of MPC1 knockout on intracellular ROS generation, MitoSox assay was performed. The intracellular ROS was found significantly higher in the MPC1−/− cells compared to the WT cells (p=0.001). To note, stimulated by hydrogen peroxide (H_2_O_2_), the intracellular ROS of the MPC1−/− cells revealed sharp increase (p<0.001), an indication of weak elimination ability of ROS in the MPC1−/− cells (Figure 5).

### MPC1−/− cells were slow-cycling and expressed higher levels of stemness genes

**Figure 6:**
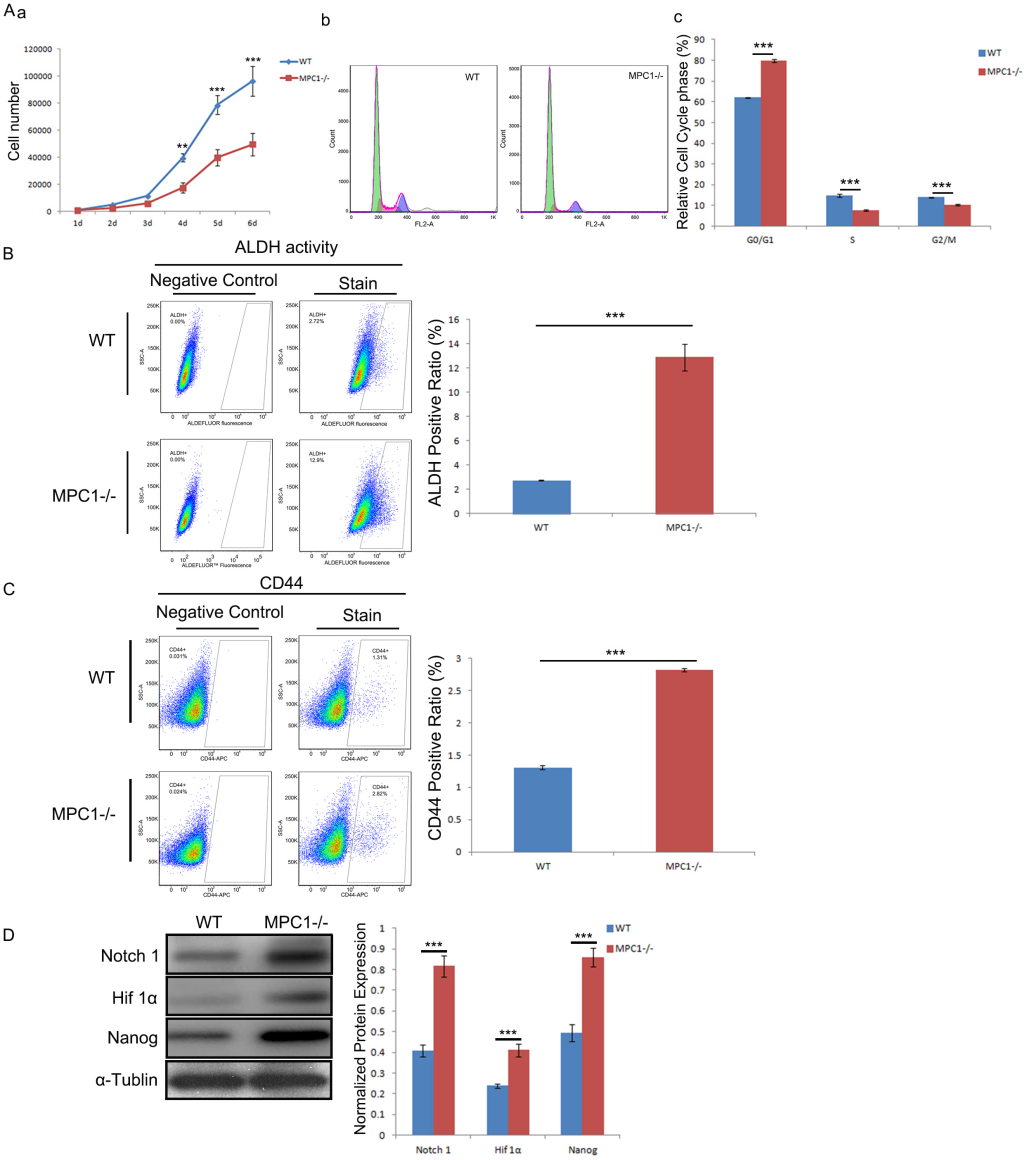
Increased cell stemness in the MPC1−/− cells **(A)** Shows the influence of MPC1 knockout on cell growth (a) and cell cycle (b). The corresponding histograms of cell cycle distribution of the MPC1−/− cells compared to the WT cells are shown in c. Representative ALDH flow cytometry figures and corresponding histograms are shown in **(B)**. Representative CD44 flow cytometry and corresponding histograms of CD44 are shown in **(C)**. Representative Western blots of Notch1, Hif 1α and Nanog and correspondingly normalized protein expression histograms are shown in **(D)**. α-Tublin was used as a loading control. ***P<0.001. Data were expressed as mean ± SEM. Three replicated experiments were carried out with the similar results.

To determine whether MPC1 knockout has a suppression effect on cancer cell proliferation, we firstly explored its influence on cell growth. Although there was no apparent effect on cell morphology, growth curves demonstrated that the growth of the MPC1−/− cells was suppressed compared with the WT cells (Figure 6Aa). For further analyses, cell cycle progression of MPC1−/− cells using PI pulse-chase was examined. As shown in Figure 6Ab and 6Ac, MPC1 knockout significantly increased the proportion of G0/G1 phase cells (p<0.001) and decreased the proportion of S (p<0.001) and G2/M phase (p<0.001) cells, indicating a G0/G1 phase arrest.

The flow cytometric detection showed a significant increase of ALDH activity in the MPC1−/− cells in comparison to the WT cells (p<0.001, Figure 6B). Similarly, flow cytometric analysis showed significantly increased CD44 (p<0.001, Figure 6C). The Western blotting analysis showed up-regulated expressions of Nanog, Hif1α and Notch1 (Figure 6D). Collectively, the findings confirm the prediction that MPC1−/− cells have markedly increased stemness features.

### MPC1−/− cells were significantly more migratory

**Figure 7:**
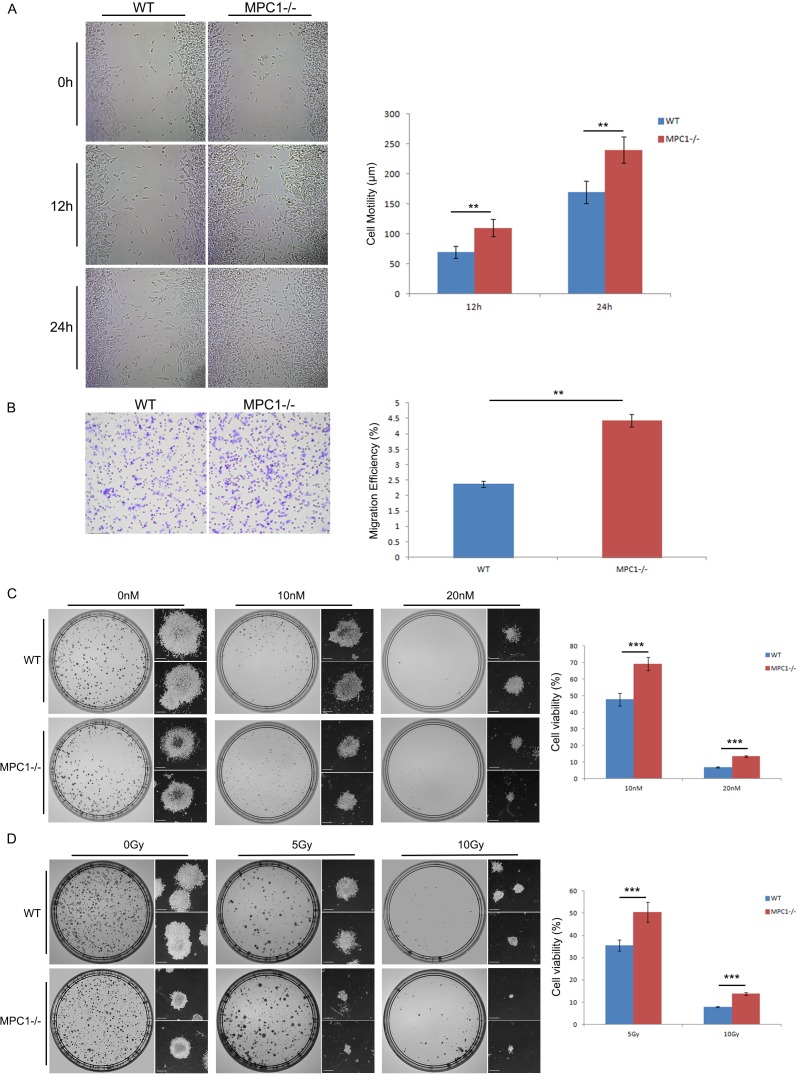
Enhanced invasion ability and therapy resistance in the MPC1−/− cells Representative wound healing images are shown on the left part of **(A)**, and the corresponding histograms of the cell mobility results are shown in the right part of the **(A)**. Representative transwell assay images are shown in **(B)**, and the corresponding histograms of the cell invasion alterations are shown on the right part of **(B)**. Representative modified colony formation assay images for chemotherapy sensitivity are shown on the left part of **(C)**, and the corresponding cell viability histograms are shown on the right part of **(C)**. Representative modified colony formation assay images for radiotherapy sensitivity are shown on the left part of **(D)**, and the corresponding cell viability histograms are shown on the right part of **(D)**. Scale bars, 500 μm. **P<0.01, ***P<0.001. All experiments were performed at least three times with consistent and repeatable results.

Wound healing assay was used to evaluate the invasion ability of the MPC1−/− cells. It was revealed that there was a significantly higher healing speed of the MPC1−/− cells at both 12 hr (p=0.001), and 24 hr (p=0.002) as shown in Figure 7A. Further transwell cell migratory assays demonstrated that the MPC1−/− cells exhibited significantly stronger migration capability compared with the WT cells (Figure 7B, p=0.003), indicating that the MPC1 gene is negatively associated with the prostate cancer cell migration and therefore plays an important role in the development of tumor invasion.

### MPC1−/− cells were significantly more resistant to both chemotherapy and radiotherapy

To evaluate whether deletion of MPC1 gene affected the sensitivity of chemotherapy, we assessed cell viability after docetaxel application in cell culture. As shown in Figure 7C, significantly more MPC1−/− cells survive with the 10 nM and 20 nM decetaxel treatments (p<0.001), reflecting significantly higher docetaxel therapy resistance in the MPC1−/− cells.

Next, we also examined the radiosensitivity of the MPC1−/− cells by the modified colony formation assay as reported earlier [18]. Compared to the WT cells, there were more MPC1−/− cells survived the 5 Gy and 10 Gy irradiations (p<0.001, Figure 7D). Microscope images showed that there were not only more clones, which were bigger, but also more living MPC1−/− cells in small clusters compared to the WT cells, indicating that the MPC1−/− cells are more resistant to irradiation.

### MPC functional blockade with UK5099 in ovarian cancer cells disclosed similar results

**Figure 8:**
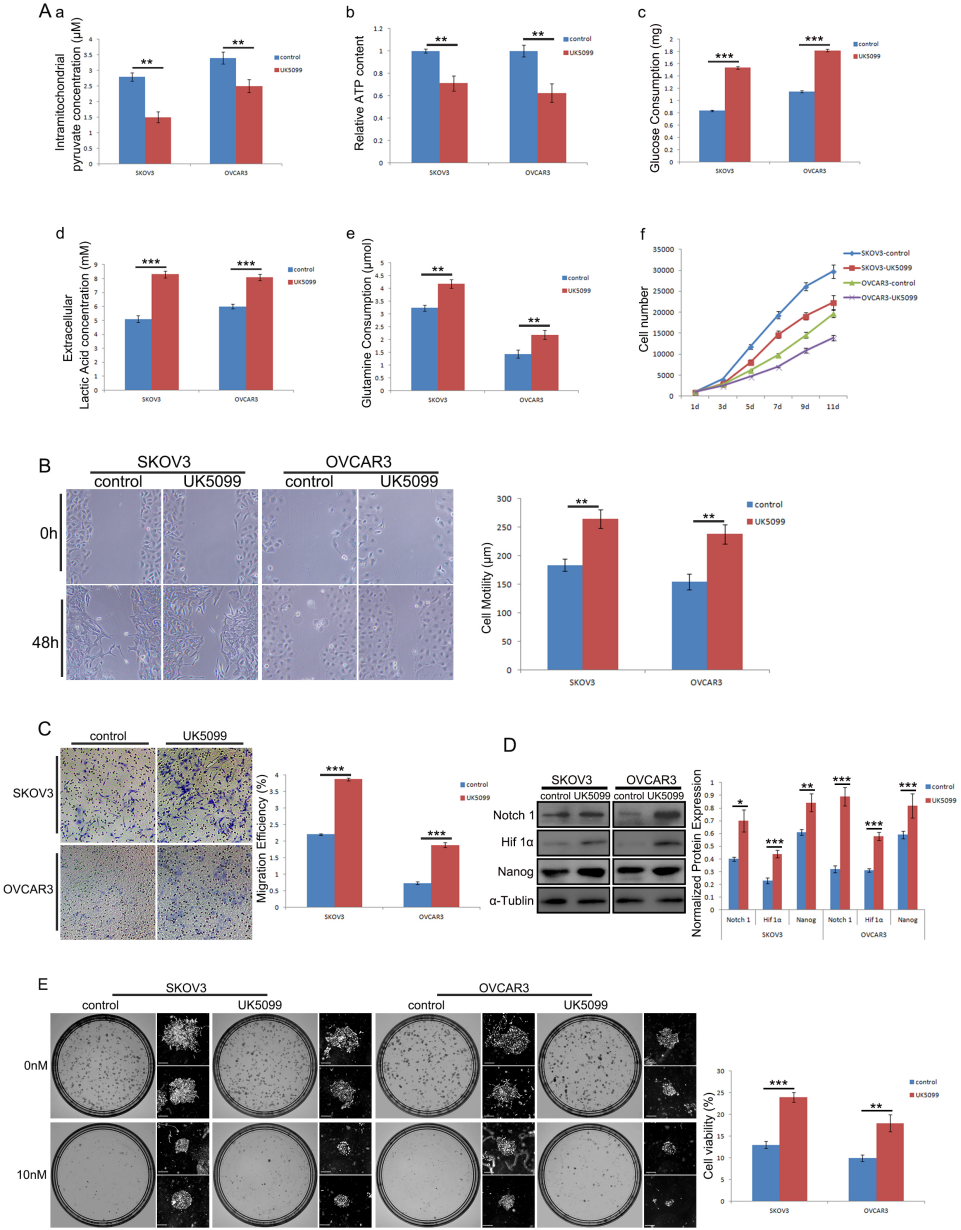
Effect of MPC blockade with UK5099 on ovarian cancer cell lines SKOV3 and OVCAR3 **(A)** Shows the results of intramitochondrial pyruvate concentration (a), ATP content (b), glucose consumption (c), lactate concentration (d) and glutamine consumption (e) when the cells were cultured with 20 nM UK5099 for one week. The effect of 20 nM UK5099 on cell growth is shown on growth curves (f). Representative wound healing images and corresponding histograms are shown in **(B)**. Representative transwell assay images and corresponding histograms are shown in **(C)**. Representative Western blots of Notch1, Hif 1α, and Nanog and correspondingly normalized protein expression histograms are shown in **(D)**. Representative modified colony formation assay images for docetaxel sensitivity and corresponding cell viability histograms are shown in **(E)**. Scale bars, 500 μm. *P<0.05, **P<0.01, ***P<0.001. Data were expressed as mean ± SEM. Three replicated experiments were carried out with similar results.

To further study the function of MPC in regulating cell stemness and metabolic reprogramming, MPC inhibitor UK5099 was applied in two human ovarian cancer cell lines SKOV3 and OVCAR3. One week after treated with 20 μM UK5099, the cells were harvested for MPC function analyses. As is shown in Figure 8A, there are significantly reduced intramitochondrial pyruvate (a, p=0.001 for SKOV3 cells, p=0.004 for OVCAR3 cells) and ATP (b, p=0.002 for SKOV3 cells, p=0.003 for OVCAR3 cells), and significantly increased glucose consumption(c, p<0.001 for both SKOV3 and OVCAR3 cells), extracellular lactate acid production (d, p<0.001 for both SKOV3 and OVCAR3 cells) and glutamine consumption(e, p=0.001 for SKOV3 cells, p=0.005 for OVCAR3 cells). In addition, the cells all grow slowly under the UK5099 application as shown in the growth curves (f). The MPC blockade with UK5099 resulted in significantly quicker wound healing as shown in Figure 8C after 48 hr wound healing tests (p=0.002 for SKOV3 cells and p=0.003 for OVCAR3 cells) and significantly more cells migrated through the chamber after 24 hr in experiments as shown in Figure 8B (p<0.001 for both SKOV3 and OVCAR3 cells). There were significantly higher expression levels of stemness markers Notch1, Hif1α and Nanog in both UK5099 treated cell lines (Figure 8D). The modified colony formation assays revealed significantly higher cell viability in both UK5099 treated SKOV3 (p<0.001) and OVCAR3 (p=0.003) when 10 nM decetaxel treatments was applied (Figure 8E).

## DISCUSSION

Main energy source from TCA cycle occurs in the mitochondria and dysfunctional mitochondrion cause diabetes, obesity and carcinogenesis [19–21]. Knowing that mitochondria is essential to metabolism, we show herein that pyruvate transport blocking via deletion of the MPC1 gene has impact on the metabolism and biological behaviors in prostate cancer cells *in vitro*.

MPC1 and MPC2 were originally known as BRP44L and BRP44 [22], which form a heterologous protein complex in the inner membrane of mitochondria [7, 8]. The intact MPC complex is about 150kd, though either of the subunit has only 15kd, which is much larger than the combination of the two subunits [23]. Human MPC1 is located at the genomic locus of 6q27 [13, 24]. In this study, MPC1 gene in the murine prostate cancer cell line RM-1 was successfully mutated by shifting mutations in the exon 1. It was verified in our study that the MPC1 gene knockout in this cell model resulted in deficient protein expression of MPC2, which is in line with the previous findings. One study in *Drosophila* demonstrated that the knockdown of the expression of CG14290 protein (MPC1) also reduced the protein levels of CG9399 (MPC2) [25]. In a mouse model study, it was disclosed that a negative MPC2 gene mutation led to marked reduction in both MPC1 and MPC2 protein expression [26]. Recently, it was also shown that MPC1 gene knockout mouse cells express neither MPC1 nor MPC2 protein [27]. In the present study, we have demonstrated the mitochondrial location of both MPC1 and MPC2 proteins in the WT prostate cancer cells by IF and Western blotting.

There are studies showing that cells are able to reprogram their metabolism toward glutamine oxidation in response to the suppression of MPC function [28, 29]. In our study, significantly decreased intercellular glutamine by GC/MS examination and significantly increased glutamine consumption by glutamine colorimetric assay were verified in the MPC1−/− cells. Moreover, upregulated expression of the glutaminolysis-related proteins (GLS and GDH) was also identified in these cells, providing a strong indication of the anaplerotic glutaminolysis.

It has been also revealed that alanine participates in the anaplerotic process when MPC genes are deleted [1, 30, 31], and the anaplerotic mitochondrial pyruvate is originated from pyruvate-alanine transamination. Indeed, we have found an increased level of ALT1 expression at protein level and significantly higher consumption of alanine in the MPC1−/− cells as verified by GC/MS analysis. Moreover, the MPC1−/− cells were more sensitive to alanine inhibitor, which indicates that pyruvate-alanine transamination pathway was activated when the pyruvate transportation was blocked. Meanwhile, we also found an overexpression of PC protein in the MPC1−/− cells. Mitochondrial pyruvate may be carboxylated by PC, an anaplerotic reaction that serves to replenish the TCA cycle with oxaloacetate. However, PC is also the initial step in a pathway called gluconeogenesis, in which glucose is synthesized from metabolites such as lactate, pyruvate or amino acids.

Given the role of mitochondrial MPC in central carbon metabolism, we have demonstrated that the MPC1 gene knockout blocks pyruvate transport into mitochondria, which was confirmed with the decreased pyruvate concentration in the mitochondria. However, the transporter deletion has not caused a complete pyruvate vanish in mitochondria. This can be explained in a way that the mitochondrial pyruvate may be generated by multi-pathways including at least the alanine transamination [1, 30, 31]. Moreover, the expression of PDHE1, which plays the main role in converting pyruvate into Acetyl-CoA, is not significantly changed in the knockout cells, indicating that the anaplerotic pyruvate production in the mitochondria keeps the PDHE1 protein expression.

Oxidative stress occurs when an imbalance between cellular antioxidant defense system and ROS appears [32]. Several studies have shown that ROS modulates the cell cycle through the oxidative stress mechanism [33]. As found in our research, the MPC1−/− cells were in slow cycle with significantly higher ROS levels. The role of ROS in mitochondrial dysfunction and abnormal cell signaling activation has been widely studied [34–36]. It is known that H_2_O_2_ can cause oxidative damage if not converted rapidly into less toxic species. The present study showed that exposed to H_2_O_2_ induces a rapid ROS production in the MPC1−/− cells, indicating a reduced ability to eliminate the toxic substances, or a reduced anti-ROS capability.

It has long been demonstrated that increased glycolysis may promote cancer progression through many ways [37, 38]. Studies show that stem cells can adjust their metabolism to preferentially glycolytic profile to produce enough amount of energy for cell growth [39]. It is in line with our present study that the MPC1 knockout forces the cells to rely on glycolysis with significantly larger amount of extracellular lactic acid secretion.

MPC1 mutations in patients are remarkably associated with severe defects in pyruvate metabolism [7, 40]. Our current study has demonstrated that MPC1 deletion in cancer cells caused a reduced cell growth and slow cell cycling in MPC1−/− cells. However, these cancer cells have obtained stronger survival ability and developed more malignant features. Thus, these cells have altered glycolytic metabolism, or Warburg metabolism. Cancer stem cells may hold the characteristics of non-proliferative state and dormancy status [41], and slow cycling MPC1−/− cells meet these criteria. To disclose the slow cycling mechanism in the MPC1−/− cells, cell cycle analysis was carried out and G0/G1 retention was verified in the cells.

Studies on low expression of MPC1 or MPC2 in embryonic stem cells have shown that MPC may modulate the pyruvate metabolism and that may be important for the maintenance of stem cell population [42, 43]. As described above, the MPC1−/− cells possess a characteristic of slow cycling, which is the characteristic of cancer stem cell under certain conditions. Most importantly, the tumor initiation capacity seems to be restricted to a small population of tumor cells that continuously maintain tumor growth [44]. Pluripotency marker Nanong was found in embryonic stem (ES) cells and Notch1 play an important role in the cell proliferation and stem cell maintenance [45–47]. The expression of these stem cell markers was found to be significantly up-regulated in the MPC1−/− cells, which is in accordance with the previous studies showing the cell stemness correlation with negative MPC expression in cancers [11, 24]. ALDH and CD44 are stem cell markers commonly used to identify cancer stem cells [48, 49]. As seen in our study, the MPC1 knockout cells have significantly higher ALDH activity and CD44 expression. All the above results indicate higher cell stemness of the MPC1−/− cells.

We next analyzed the chemosensitivity and radiosensitivity alterations and found that the MPC1−/− cells were more resistant to both therapies compared with the WT cells. These data strongly suggest that the metabolic shift caused by the MPC1 knockout is related to cell stemness up-regulation. We further found that the MPC1−/− cells significantly increased glycolysis and cell invasion, which is consistent with the negative correlation between MPC1 expression and patient survival rate [14, 50], indicating its tumor genetic role in prostate cancer.

To verify the role of MPC in cell stemness and metabolic reprogramming in other human cancer cells, we extended our studies by using MPC inhibitor UK5099 in human ovarian cancer cell lines SKOV3 and OVCAR3. It was discovered that both cell lines under 20 μM UK5099 application showed MPC function inhibition as revealed significantly less intramitochondrial pyruvate and ATP production, and significantly higher levels of glucose and glutamine consumption and extracellular lactate production. Correspondingly, the UK5099 blockaded ovarian cancer cells showed significantly higher migratory and wound healing abilities, in addition to significantly higher docetaxal resistance. Meanwhile, the UK5099 treated ovarian cancer cells expressed significantly higher levels of stamness markers, which all are similar to the MPC1 knockout cells.

In conclusion, our results have proved that the MPC1 knockout blocks mitochondrial pyruvate uptake and OXPHOS, which results in metabolic reprogramming towards aerobic Warburg glycolysis with reduced ATP production capability. Moreover, it has also shown that there are significantly activated anaplerotic glutamine and alanine TCA cycle pathways in the MPC1−/− prostate cancer cells. In parallel with the above mentioned Warburg effect, the MPC1−/− cells show significantly higher stemness reflected by substantially higher expression levels of several stemness factors, highly migratory behavior and resistance to both chemotherapy and radiotherapy. MPC inhibitor UK5099 in human ovarian cancer cell lines SKOV3 and OVCAR3 resulted in similar results. Therefore, the current study reveals an important role of MPC in the determination of metabolic reprogramming and cell stemness in cancer cells.

## MATERIALS AND METHODS

### MPC1 knockout cell line establishment

The mouse prostate cancer cell line RM-1 was provided by Beijing Nuolanxin Bio-Chemistry Co. Ltd. Two plasmids containing the MPC1-Cas9/sgRNA vectors targeting to the mouse MPC1 exon1 (Beijing Nuolanxin Bio-Chemistry Co. Ltd, China) were applied in this study. The sequences of the sgRNA in the plasmids are shown below:

MPC1-g1: ggcggactatgtccggagcaAGG

MPC1-g2: gcaaagcggcggactatgtcCGG.

When grown in 60%-70% confluent, the cells were transfected with the plasmids composed of sgRNA, Cas9 and anti-puromycin gene, and 10 μl lipofectamine 2000 (Invitrogen, 11668019) in 3 ml serum-free medium. At 24 hr after transfection, the medium was changed with fresh containing 10% FBS. The cells were cultured for 24 hr under ordinary culture condition before the medium was replaced with fresh medium containing puromycin for 72 hr. The cells were then harvested and single cell suspension in 1 cell/100 μl was made by limiting dilution. 100 μl/well cell suspension was cultured in 96-well plate for 2 weeks for cell cloning. Monoclonal cells were obtained after two rounds of such cloning. All the cells were cultured in 10% fetal bovine serum (FBS, Gibco^™^, 10500-064) of RPMI 1640 medium (Gibco^™^, 11835-063) supplemented with 100 U/ml of penicillin and 100 μg/ml streptomycin (Life Technologies, 15140122) at 37°C with 5% CO_2_. MTT was used for proliferation assay.

### Mutation analysis

The MPC mutation identification was performed with DNA extracted according to the manufacturer's protocol (OMEGA, D3396). The DNA was then used for PCR amplification with PCR reaction mixes contained 10 μl Taq Master Mix (CWBIO, CW0682), 1 μl of each primer (Forward: AGCGGTCGTAAGGCTTCTCC, Reverse: TCCCCTGAGTCCTGCTGTCC), 2 μl of DNA template and RNase-Free water in final volume of 20 μl. Cycling conditions included an initial hot start at 94°C for 30 sec, 40 cycles of 94°C /25 sec, 60°C /20 sec and 72°C /25 sec, plus a final 72°C extension for 10 sec. The PCR products were subjected to sequencing by Sangon Biotech.

### Western blotting

For whole cell protein extract preparation, cells were dissolved (for 30 min) in lysis buffer containing 1% PMSF in RIPA buffer (Thermo Scientific, West Palm Beach, FL) before centrifuged at 12,000 rpm for 5 min at 4°C. The cytoplasmic and mitochondrial proteins were extracted according to the instructions provided together with mitochondria/cytosol fractionation kit (Biovision, K256-25).

30 μg proteins were loaded onto a 10% SDS-PAGE and transferred to polyvinylidene difluoride transfer membrane (PVDF). Membranes were incubated with the indicated antibodies overnight, followed by the incubation with the corresponding secondary antibodies conjugated with horseradish peroxidase-conjugated (HRP). Finally, the membranes were detected by enhanced chemiluminescence (Amersham, Arlington Heights, IL) and exposed to X-ray film. The intensity of band was normalized to α-Tubulin antibody (sigma, T9026) and analyzed using Image Lab 2.0 Software (Bio-Rad Laboratories Inc, USA). The primary antibodies used in this study were as follows: MPC1 (NBP1-91706), GLS (NBP2-29940), GDH (NBP2-16679) and PC (NBP1-49536) were bought from NOVUS. Notch 1 (3608), MPC2 (46141), and PDHE1 (3201) were purchased from Cell Signaling Technology. Nanog (AF1997) and Hif1α (AF1935) were from RD System. ALT1 (ab202083) was from abcam.

### IF and ICC detection

Cells were seeded in 35-mm glass bottom plates at a density of 1×10^5^ and cultured for 48 hr. Then cells fixed with methanol for 10 min and treated with 0.1% Triton for 10 min, followed by incubation in 3% BSA-PBS solution for 15 min. After that, the cells were stained by MPC1 or MPC2 and MT-CO1 (abcam, ab14705) for 2 hr. For IF detection, samples were incubated with corresponding second antibody for 30 min. The cells were visualized by a Leica fluorescence imaging system. While doing the ICC detection, HRP was used to incubate for 30 min. Plates were then stained with 3, 3′-diaminobenzidine tetrahydrochloride (DAB, Dako) for 10min, counter-stained with hematoxylin and dehydrated.

### Flow cytometry analysis

Cell suspensions of 1×10^6^ cells/ml were used for flow cytometry analysis. For cell cycle analysis, cell suspensions were fixed by ice-cold 75% ethanol in -20°C overnight. Then cells were stained with 2 μg/ml RNase A and 10 μg/ml PI in 500 μl PBS. For ALDH activity assay, 500 μl of ALDEFLUOR^™^ Assay Buffer containing 5 μl of activated ALDEFLUOR^™^ reagent was used to resuspend the cells for 45 min at 37°C. Following incubation, all tubes were centrifuged for 5 min at 600 rpm. Centrifuged cell pellets were resuspended in 500 μl of ALDEFLUOR^™^ Assay Buffer. For CD44 stain, 15 μl of CD44 antibody (BD Pharmingen, 559942) dissolved in 1 ml FluoroBrite™ DMEM medium was added to cell suspension and cultured for 45 min at 37°C. Intracellular ROS production was detected using MitoSOX^™^ Red (MitoSox, Invitrogen). The cells were incubated with MitoSox at a concentration of 5 μM for 30 min at 37°C. H_2_O_2_ (100 μM) was used to stimulate ROS accumulation in cells. Samples were analyzed on a BD LSRII flow cytometer. Software FlowJo version 7.6 was used for further data analysis.

### Measurement of mitochondrial pyruvate concentration

Cells were harvested at 80% confluent and 5×10^7^ cells were left for mitochondria isolation. Manipulate according to the first part of mitochondria/cytosol fractionation kit (Biovision, K256-25). 50 μl lysis buffer was added to the isolated mitochondria and homogenized by sonication. Pyruvate concentration was determined by pyruvate colorimetric assay kit (BioAssay, K609-100). Briefly, 90 μl working reagent (including enzyme mix and dye reagent) was added to each 10 μl of sample or standard before. Tap plate to mix and incubated 30 min at room temperature. Samples as well as standards absorbance were measured at OD570nm.

### Glucose uptake assay

The glucose uptake was detected by glucose assay kits (Sigma-Aldrich, GAHK-20). Cells were seeded in 6-well plate and cultured for 12 hr and 24 hr before medium collection. Then the media were measured using a Biochrom Assays UVM340 Microplate Reader. Data were normalized based on the viable cell counts measured by cell counting.

### Lactate assay

Cells were seeded evenly into 6-well plate and cultured for 12 hr to be adherent to the bottom before the same volume of 3 ml medium were replaced and cultured for 24 hr. Then the cell culture medium was centrifuged and ready to go lactate assay. At the same time cells were harvested in 200 μl PBS and cell lysis was achieved by sonication (in ice-water bath). Transfer the clear supernatant to go lactate assay before centrifuging homogenate at 12,000 rpm for 10 min. The lactatic acid concentration in the 3 ml culture medium and 200 μl cell lysis was examined by the lactate assay using an EnzyChrom^™^ l-lactate assay kit (Bioassay Systems, ECLC-100). Briefly, add 20 μl samples in 96-well plate followed by adding 80 μl working reagent (contained lactate dehydrogenase, NAD, MTT) per reaction well quickly. Read optical density (OD) of OD_0_ for time zero and OD_20_ after a 20 min incubation at room temperature. The sample lactate acid concentration was determined by OD values from the standard curve. Data were normalized based on the viable cell counts measured by cell counting.

### L-glutamine measurement

The concentrations of L-glutamine in culture media were determined (Glutamine Colorimetric Assay Kit, Biovision, K556-100). Briefly, cells were seeded in 6-well plate and allowed to adhere overnight. Changed with the fresh medium and cultured for 12 hr and 24 hr, supernatant was achieved by centrifuge at 1000 rpm for 5 min. 40 μl sample supernatant with 60 μl reaction buffer was added to 96-well plate. After 1 hr incubation at 37°C, samples as well as standards absorbance were measured at OD450nm. Data were normalized based on the viable cell counts measured by cell counting.

### GC/MS analysis

1×10^7^ cells were collected in ice-cold methanol and prepared with rapid quenching in liquid nitrogen. Cellular debris was pelleted by centrifugation, and the supernatant was dried by vacuum centrifugation prior to resuspension in 50% acetonitrile and analysis by GC/MS. Targeted metabolic analysis were performed on an Agilent 7890 gas chromatograph system coupled with a Pegasus HT time-of-flight mass spectrometer. Chromatographic separation was performed on a DB-5MS capillary column (J&W Scientific, Folsom, CA, USA). Helium was used as the carrier gas. The MS data were acquired in full-scan mode.

In the metabolomics analysis, nine independent pairwise comparisons were performed. All data was imported into SIMCA14 software (Umetrics, Umea, Sweden) for PCA and OPLS-DA. To refine this analysis, the first principal component of variable importance projection (VIP) was obtained. The VIP values exceeding 1.0 were first selected as changed metabolites. In step 2, the remaining variables were then assessed by Student's T test.

### Kinetic measurement of cytotoxicity

The effect of inhibitors on cell survival ability was analyzed using an IncuCyte ZOOM^™^ Imaging System and image analysis software (Essen BioScience Inc.). Briefly, 5000 cells/well were seeded in 96-well plates and incubated with different concentrations of β-CH inhibitor (Sigma, 51887-89-9). Annexin V Red (Essen BioScience Inc., 4641) was diluted as the manufactures` instruction and was used to stain the cells for detection of apoptosis. Red object confluence (%) was used to represent apoptosis ratio.

### OCR and ECAR analyses

OCR and ECAR were performed using a Seahorse XF^e^96 analyzer (Seahorse Bioscience, USA). Cells were plated at a density of 2×10^4^ cells/well in XF^e^96 cell culture microplate and allowed to attach overnight. Growth medium was removed from each well and changed to analysis medium supplemented with 4 mM glucose, 2 mM sodium pyruvate and 2 mM glutamine. Cells were then maintained at 37°C in non-CO_2_ incubator for 1 hr prior to the measurements of metabolic rates. OCR and ECAR were detected under basal conditions followed by the sequential injection of oligomycin (1 μM), FCCP (1 μM) and rotenone/antimycin A (0.5 μM). The obtained results were corrected by cell number, which was estimated by nuclear staining with Hoechst.

### Wound healing and migration assay

Cells were seeded in 6-well plate and cultured for 16 hr for adherence. Scratches were made by 10 μl pipette tips and washed twice with PBS. 2 ml serum-free medium was added to the plate. The wound width was measured after 12 hr, 24 hr and 48 hr. The average scratch width was defined by using Adobe^®^ Photoshop^®^ CS6 software. For migration assay, cells in 200 μl serum-free medium were added to the upper chamber with 8 μm pore (Corning, USA) and 700 μl medium with 10% FBS was added in the bottom chamber. Cells were migrating at 37°C in 5% CO_2_ for 14 hr or 24 hr. The filters were then fixed in methanol and stained with crystal violet for 20 min, respectively. Migrated cells on the membrane were counted under a microscope.

### Chemosensitivity and radiosensitivity assays

For chemosensitivity assay, cells were plated into 60-mm dish and cultivated for 12 hr before the cells were attached. Then different concentrations (10 nM and 20 nM) of docetaxel (Sigma) were added to the medium for 2 weeks culturing before the cells were stained with crystal violet. For radiosensitivity assay, cells were seeded into 60-mm dish and incubated overnight for cells adherence. Then 5 Gy and 10 Gy X-ray irradiations were performed onto all of the dishes. The cells were kept for cultivation for 2 weeks before colony formation ability was evaluated. During the cultivation period, the medium was changed every three days. Two weeks later, the cells were fixed with 4% paraformaldehyde (PFA) and visualized by staining with 0.1% (w/v) crystal violet in methanol. The dishes were then washed and dried before the colonies were counted in a G: BOX F3 multifunction imaging system with related software (Syngene).

### MPC blockade experiments with UK5099 in human ovarian cancer cell lines SKOV3 and OVCAR3

The SKOV3 and OVCAR3 ovarian cancer cell lines were purchased from American Type Culture Collection (ATCC) and routinely maintained in our lab according to the ATCC recommendation. Briefly, the cells were cultured in 10% fetal bovine serum (FBS, Gibco^™^, 10500-064) of RPMI 1640 medium (Gibco^™^, 11835-063) supplemented with 100 U/ml of penicillin and 100 μg/ml streptomycin (Life Technologies, 15140122) at 37°C with 5% CO_2_. Dose-dependent tests of UK5099 ranging from 10 μM to 100 μM UK5099 in these cell lines were performed and it was discovered that 20 μM UK5099 was optimal for further experiments.

### Statistics

Results shown are representative of at least three independent experiments. Repeated measures were analyzed by one-way ANOVA and Student's T test using SPSS 17.0. Results are shown as mean ± SEM, p<0.05 was considered to be statistically significant.
